# Arterial Divestment and Resection in Post-neoadjuvant Pancreatic Adenocarcinoma

**DOI:** 10.7759/cureus.20275

**Published:** 2021-12-08

**Authors:** Yugal Limbu, Sujan Regmee, Roshan Ghimire, Dhiresh Kumar Maharjan, Prabin Bikram Thapa

**Affiliations:** 1 Department of Gastrointestinal and General Surgery, Kathmandu Medical College Teaching Hospital, Kathmandu, NPL

**Keywords:** neoadjuvant therapy, whipple's procedure, pancreatic adenocarcinoma, sub-adventitial divestment, periarterial divestment

## Abstract

Introduction

The advent of neoadjuvant therapy in the management of pancreatic adenocarcinoma has significantly improved the prognosis of the disease. Nevertheless, the only chance of long-term disease-free survival in pancreatic cancer is achieved with complete tumor resection, and artery involvement by the tumor is one of the major determinants in its resectability. We aim to evaluate the feasibility of a novel technique, namely, the periarterial divestment, which has allowed surgeons to clear the tumor tissues off the visceral arteries without the need for arterial reconstruction.

Materials and methods

In this single-center, retrospective, descriptive, cross-sectional study done between August 2019 and July 2021, seven consecutive patients with histologically confirmed pancreatic ductal adenocarcinoma (PDAC) who underwent neoadjuvant therapy were included. Arterial divestment was performed in six of seven patients and arterial reconstruction was performed in one of the patients. The data on perioperative and the early oncological outcome were recorded.

Results

Five patients underwent periarterial divestment, one underwent sub-adventitial divestment, and one underwent superior mesenteric artery reconstruction due to deeper tumor infiltration into the arterial wall. The intraoperative frozen section of periarterial tissue was positive in three cases and the final histopathological specimen after the divestment showed a positive margin in two of the cases. The clinically significant postoperative pancreatic fistula was noted in two patients, and one patient experienced grade C post-pancreaticoduodenectomy hemorrhage due to a hepatic artery pseudoaneurysm. Four patients, all of whom underwent periarterial divestment, experienced postoperative diarrhea. There were no mortality and the median postoperative hospital stay was seven days.

Conclusion

The need for arterial reconstruction in borderline and locally advanced pancreatic cancer can be avoided by using the periarterial divestment technique. Divestment of arteries is technically feasible and can be carried out safely without compromising the patient's oncological outcome. However, further validation of this technique must be done by well-designed studies with a greater sample size.

## Introduction

Despite medical and technological advances in cancer therapy, the burden of pancreatic cancer death is ever increasing worldwide [1]. Although systemic chemotherapy has been established as an essential element in managing pancreatic adenocarcinoma, complete surgical resection of the tumor is the only modality that gives the patient a chance of complete cure or long-term survival [2,3]. In the past, patients with locally advanced pancreatic ductal adenocarcinoma (PDAC) were considered not to benefit from surgery [4-6]. However, the advent of modern neoadjuvant chemotherapy has increased the prospect of these tumors to be amenable to curative resection and the overall oncological outcome has been shown to improve in the patients who undergo surgery following neoadjuvant treatment [7]. Tumor margin involvement is an independent variable contributing to recurrence, especially after neoadjuvant therapy [8]. An aggressive local dissection rather than local arterial resection can achieve a negative microscopic tumor margin [9]. Nevertheless, obtaining microscopic tumor-free margin during curative surgical resection of these tumors remains challenging, especially in this setting [10,11].

The desmoplastic reaction around the tumor and its adjacent vessels, especially after neoadjuvant therapy, mimics the active tumor infiltration of the vessels in imaging studies despite active tumor cell destruction. Arterial and venous resection and reconstruction have therefore been discussed in the literature to overcome this situation and get a tumor-free resection margin [12-15]. In 2016, Miao et al. described a technique known as arterial divestment, which has been shown to facilitate tumor-free margin without vascular reconstruction. This technique of radical periarterial soft tissue and neuro-lymphatic clearance has further been described in detail by Diener et al. from the University of Heidelberg and has shown promising early results [9]. We describe our experience using this technique in post-neoadjuvant pancreatic adenocarcinoma and its early postoperative outcome.

## Materials and methods

This study was a retrospective descriptive study of seven consecutive patients who underwent neoadjuvant therapy for histologically confirmed PDAC and were treated at Kathmandu Medical College Teaching Hospital from August 2019 to July 2021. The medical records and operative notes were reviewed for demographic, intra-, and postoperative data and pathological variables of all the patients who underwent a Whipple's procedure for post-neoadjuvant pancreatic adenocarcinoma. Postoperative complications including postoperative pancreatic fistula (POPF), delayed gastric emptying (DGE), postoperative hemorrhage (PPH), and chyle leakage were defined and graded according to the recommendations from the International Study Group of Pancreatic Surgery (ISGPS) [16]. Preoperative physical status was graded according to the American Society of Anesthesiologists (ASA) guidelines. When available, preoperative contrast-enhanced computed tomography (CECT) scan images were obtained from patients on their follow-up. Informed consent was obtained from all patients for the utilization of their data for research purposes.

**Figure 1:**
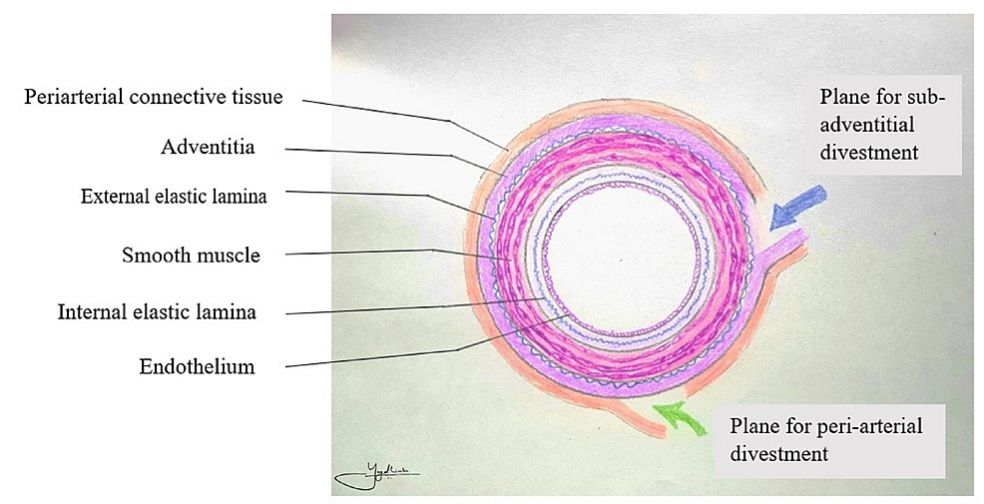
Schematic diagram showing the plane of dissection of different divestment techniques.

**Figure 2:**
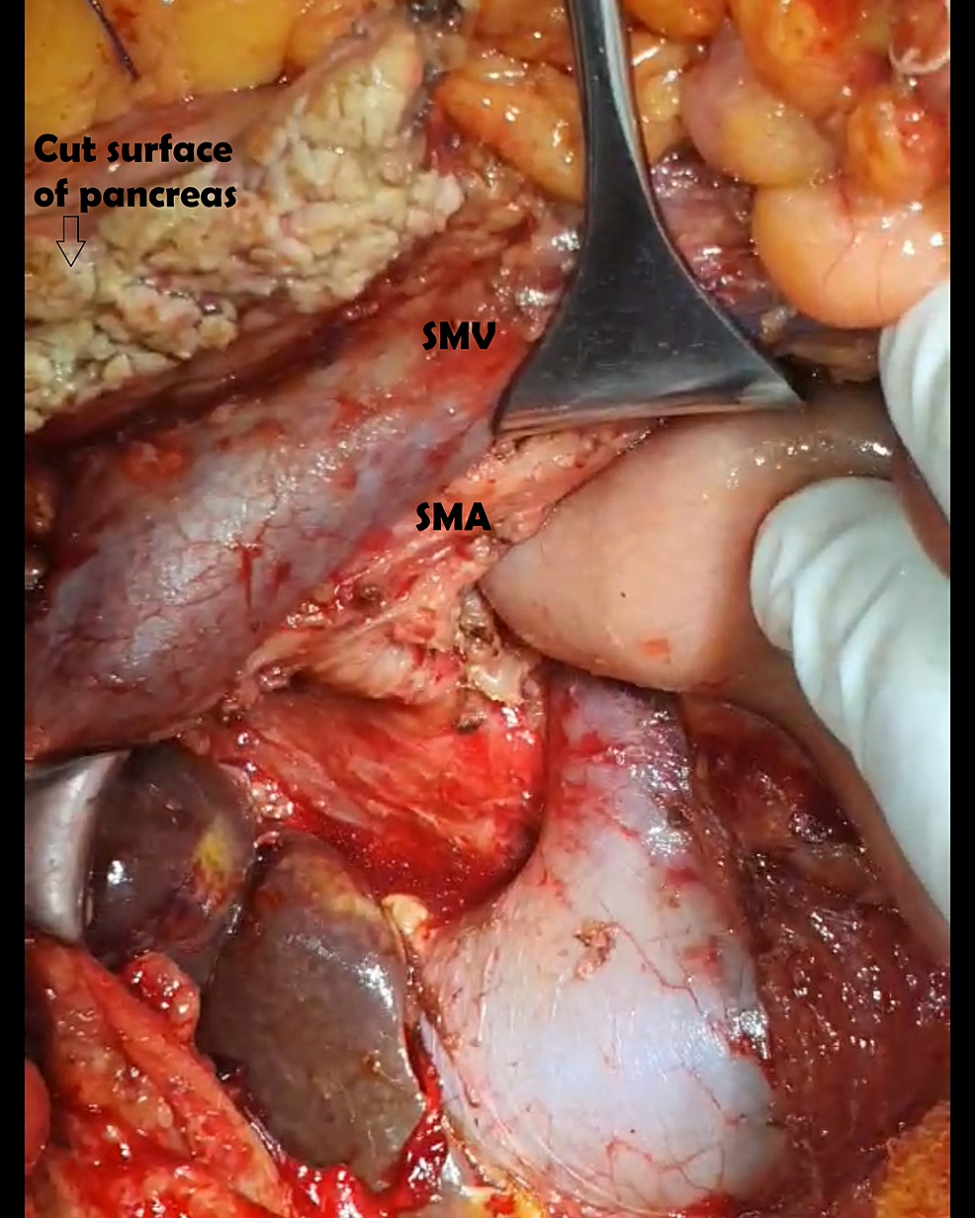
Periarterial divestment of superior mesenteric artery. SMV: superior mesenteric vein; SMA: superior mesenteric artery.

**Figure 3:**
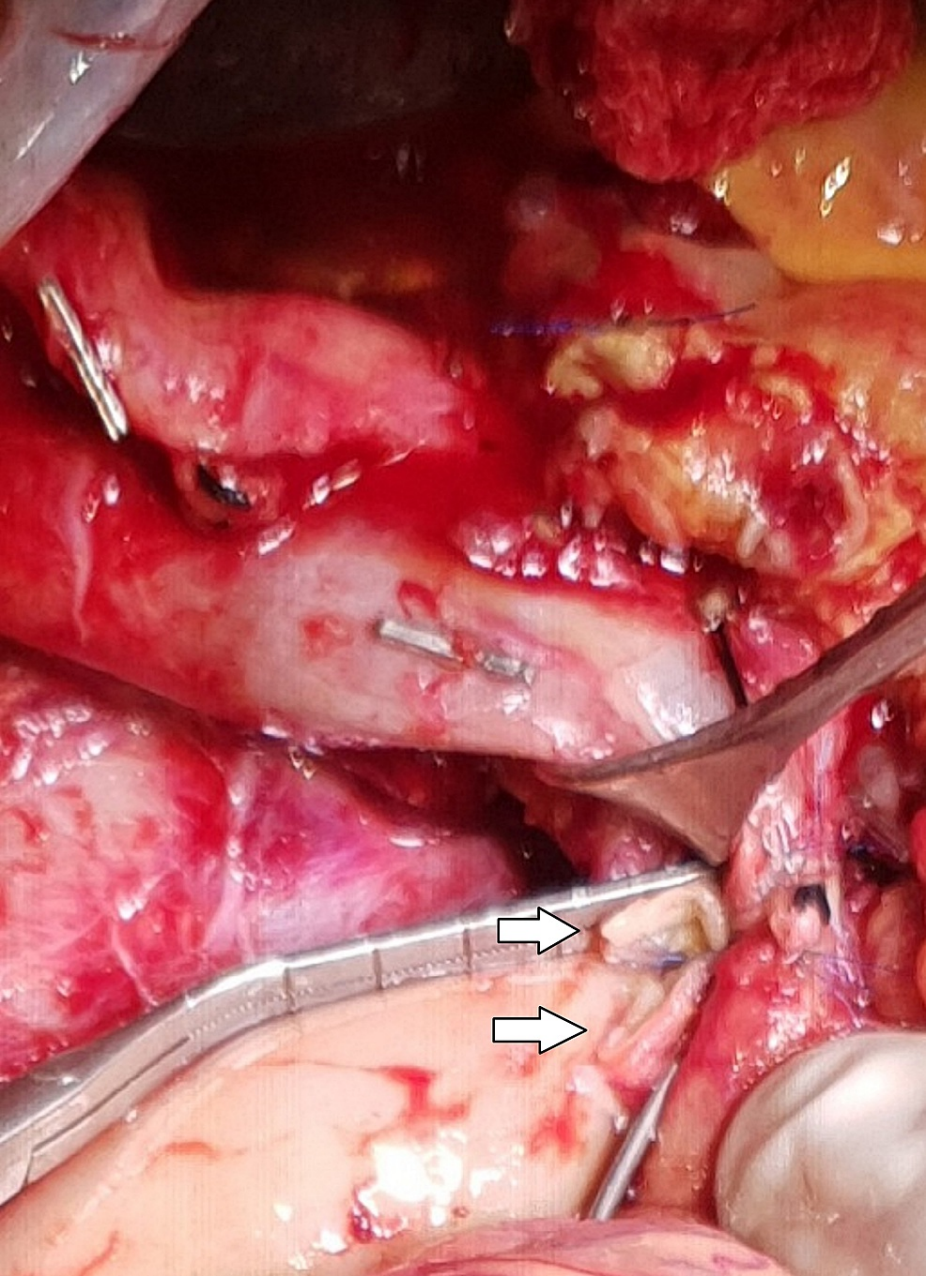
Resection of the part of the superior mesenteric artery involved by tumor. White arrows indicate the cut ends of the superior mesenteric artery under vascular clamps.

**Figure 4:**
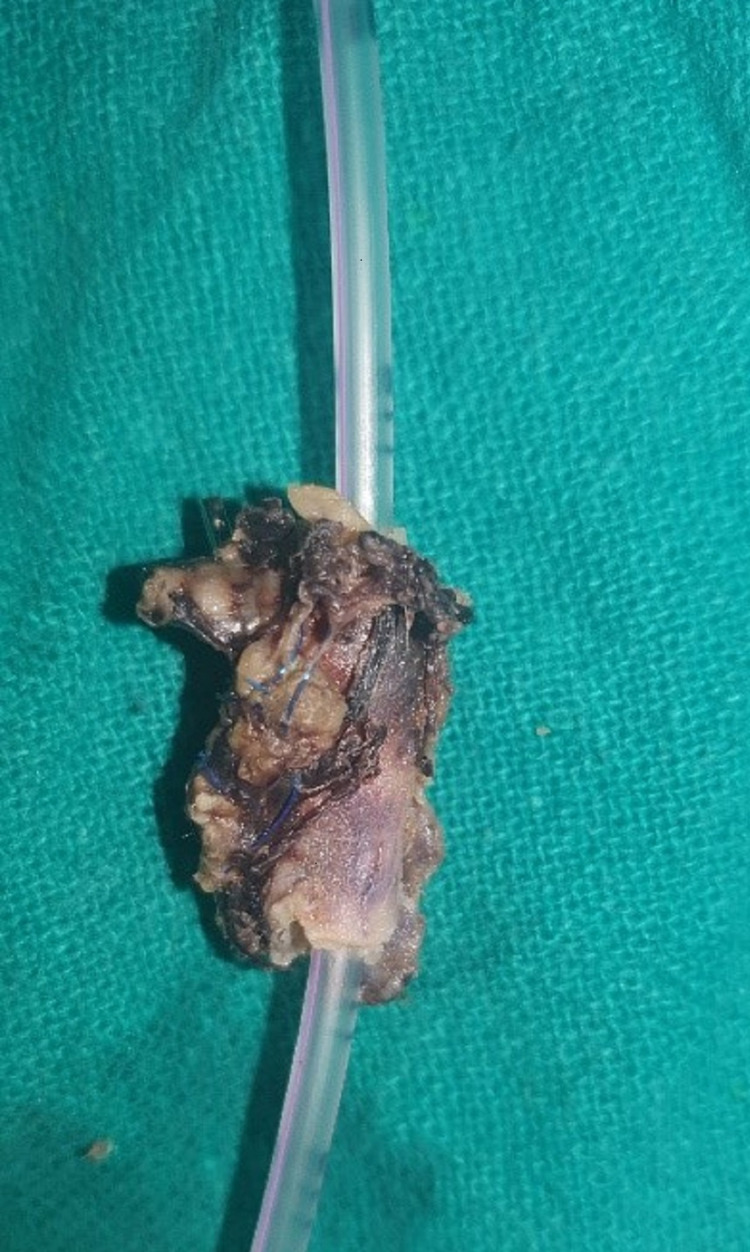
Formalin-fixed specimen of the resected part of the superior mesenteric artery.

The artery-first approach was used in all patients undergoing the periarterial and sub-adventitial divestment technique [17,18]. Frozen section sampling was done from the suspected periarterial tissue before undertaking the divestment procedure. For the periarterial divestment technique, all the soft tissue and neuro-lymphatic tissue were dissected off the tunica adventitia of the involved artery using a combination of blunt and sharp dissection as well as energy devices such as bipolar cautery and harmonic scalpel. In the sub-adventitial technique, as described by Cai et al. [16], the plane between the tunica adventitia and the white glossy external elastic lamina was identified at the arterial segment proximal or distal to the area of tumor involvement, and the dissection was carried along the plane above the external elastic lamina towards the tumor from either side (Figure 1). This plane was developed longitudinally and circumferentially until the artery was freed from any tumor (Figure 2). In instances where the dissection plane could not be clearly developed, the dissection was stopped, and artery resection and reconstruction were considered as performed in one of our cases (Figures 3, 4).

## Results

The mean age of the patients in this series was 54.4 years, and four of the seven patients were male. Distant metastasis was ruled out in all patients by cross-sectional imaging of the chest and abdomen (either CECT or contrast-enhanced magnetic resonance imaging) after the neoadjuvant therapy. The mean maximum tumor dimension on cross-sectional imaging was 3.3 cm. Five of the patients had superior mesenteric artery (SMA) encasement, and two patients had common hepatic artery encasement. Among the seven patients, two patients had undergone endoscopic retrograde cholangiopancreatography (ERCP) and biliary stenting for high serum bilirubin. Periarterial divestment was performed in five patients, and sub-adventitial divestment was performed in one patient. In addition, one patient underwent arterial (SMA) reconstruction following a failed sub-adventitial divestment procedure due to deeper tumor infiltration into the arterial wall. The perioperative outcome is shown in Table 1.

**Table 1:** Perioperative outcome of patients undergoing arterial divestment procedures. ASA: American Society of Anesthesiologists; PAD: periarterial divestment; SAD: sub-adventitial divestment; Art Recon: arterial reconstruction; Op. time: total operative duration; EBL: estimated blood loss; POPF: postoperative pancreatic fistula; DGE: delayed gastric emptying; PPH: post-pancreaticoduodenectomy hemorrhage.

Case	ASA	Operative procedure	Op. time (min)	EBL (ml)	POPF grade	DGE grade	PPH grade	Chyle leak	Diarrhea	Intraoperative frozen section	Final R status
1	1	PAD	200	200	A	A	A	-	Yes	+	R_0_
2	2	PAD	230	300	A	A	A	-	Yes	+	R_0_
3	1	PAD	270	220	B	B	A	-	No	−	R­_1_
4	2	PAD	210	330	A	A	A	-	No	−	R_0_
5	2	PAD	260	400	A	A	A	-	Yes	+	R_1_
6	1	Art Recon	350	600	B	B	A	-	No	−	R_0_
7	2	SAD	270	300	A	A	C	-	No	−	R_0_

Intraoperative frozen section from suspicious periarterial tissue was taken in all cases and was positive in three out of seven cases. Divestment or reconstruction was done in all cases regardless of the frozen section results. Perioperative blood transfusion was required in three of the patients. Two patients developed clinically significant POPF, and the same two patients developed grade B delayed gastric emptying (DGE). In one of the patients who underwent sub-adventitial divestment, grade C post-pancreaticoduodenectomy hemorrhage (PPH) secondary to hepatic artery pseudoaneurysm occurred who was successfully managed with gel foam embolization.

The final histopathological specimen showed T4 staging in all of the cases. Four of them were moderately differentiated adenocarcinoma, whereas three were poorly differentiated adenocarcinoma. All cases had perineural tumor invasion. Regarding nodal status, five patients had N1 status, and two patients had N2 status. Positive margins were noted in two cases, and among them, the frozen section was positive in one and negative in another. Three out of seven patients, all of whom underwent periarterial divestment, experienced postoperative diarrhea.

The median postoperative hospital stay was seven days and ranged from six to 14 days.

## Discussion

**Figure 5:**
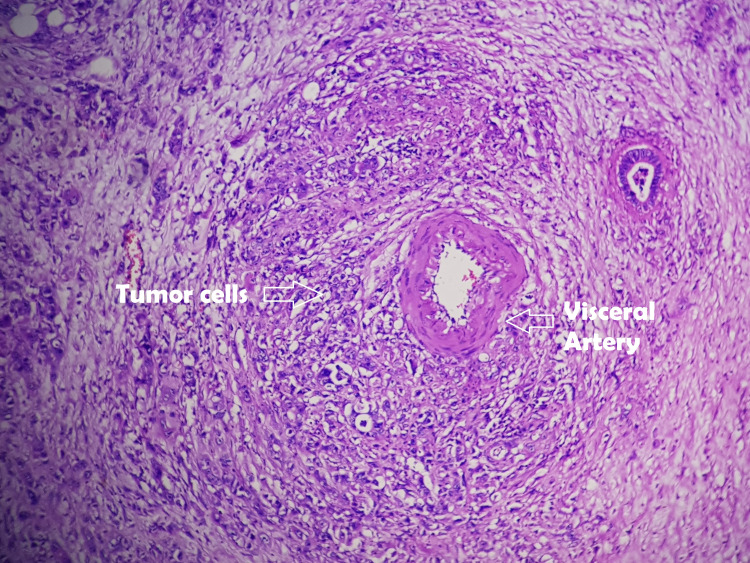
Tumor cells encasing the visceral artery without breach of the arterial adventitial layer.

**Figure 6:**
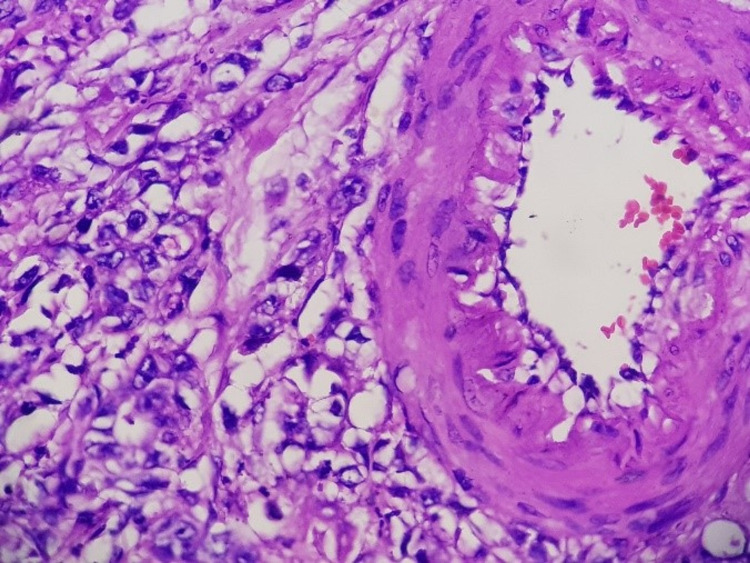
Magnified image of the tumor cells just outside the adventitial layer.

In this series, all patients received gemcitabine-based neoadjuvant therapy for locally advanced and borderline resectable PDAC. Preoperative imaging was thoroughly reviewed to rule out any distant metastasis before any surgery with curative intent. Periarterial and sub-adventitial divestment were possible in six out of seven patients, of which two patients had positive R status in the final histopathological report. As mentioned in recent literature, complete tumor removal can be achieved by either arterial resection or by arterial "divestment" [9,16]. During the divestment of an involved artery, the periarterial neuro-lymphatic tissues are dissected off the arterial wall without any vascular resection. This is particularly of interest after neoadjuvant therapy because the arterial wall often remains unaffected by active tumor cells (Figures 5, 6), and hence a radical tumor clearance can be achieved without arterial resection [9].

Arterial resection and reconstruction during Whipple's procedure for pancreatic cancer are still debated as these procedures are associated with high morbidity and mortality [9]. Moreover, the oncological benefit from arterial reconstruction has not been convincingly demonstrated in the literature [9,14,16,19]. Due to advances in neoadjuvant regimens, however, the "unresectable" tumors are being down-staged effectively in some cases [20]. The viable or devitalized tumor mass encasing adjacent arteries cannot be differentiated adequately on cross-sectional imaging; hence, it is imperative to assess these tissues intraoperatively before deciding definitive operative management. Likewise, the surgeon must decide to either divest or resect the visceral artery intraoperatively because the preoperative cross-sectional imaging cannot differentiate vascular encasement from true arterial wall infiltration in all cases [20]. In this note, the first question that needs to be answered with a high degree of certainty is when true arterial wall infiltration is absent, will arterial divestment achieve R0 resection rate as much as when arterial resection is performed. Furthermore, if it does, will that translate to improvement in the overall oncological outcome?

As seen in the result of this series, albeit a small one, arterial divestment was possible in the majority of the cases. In one of the cases, it was apparent during divestment that the tumor had infiltrated the arterial wall; hence, resection of the segment of SMA with end-to-end anastomosis was performed. Three patients in our study developed diarrhea in the postoperative period, which was controlled with loperamide. A similar result was noted in the study done by Inoue et al., in which the majority of patients undergoing periarterial neuro-lymphatic tissue clearance experienced severe diarrhea [21]. Although the exact mechanism of this postoperative diarrhea is unclear, it is mainly attributed to the circumferential dissection of the nerve plexus around the SMA [22]. Pseudoaneurysm and bleeding are also the concerns associated with arterial divestment [9]. In our series, a patient who underwent sub-adventitial divestment developed a hepatic artery pseudoaneurysm whose bleeding was controlled by interventional radiology. Nevertheless, studies have shown that the divestment technique is safe and without excessive mortality, especially when compared to arterial resection and reconstruction [16,23].

Regarding the intraoperative frozen section, it has been discussed in the literature that false-negative findings are likely because frozen sections cannot analyze the entire suspicious area [9]. As observed in one of our cases, the final histopathological report showed a positive tumor margin even though the frozen section was negative. Hence, frozen sections cannot be entirely relied upon, and divestment should be advocated whenever clinical suspicion arises in such a situation.

The limitation of our study is its small sample size and the lack of long-term oncological outcomes.

## Conclusions

The need for arterial reconstruction in borderline and locally advanced pancreatic cancer can be avoided by using the periarterial and sub-adventitial divestment techniques. Divestment of arteries is technically feasible and has shown early oncological outcomes comparable to arterial resection and reconstruction. However, surgeons should not rely on the intraoperative frozen section to determine the indication of arterial divestment.
